# Supplementary material for: The predatory soil bacterium Lysobacter reprograms quorum sensing system to regulate antifungal antibiotic production in a cyclic-di-GMP-independent manner
Source: Commun Biol. 2021 Sep 24;4:1131. doi: 10.1038/s42003-021-02660-7 (PMC8463545; doi:10.1038/s42003-021-02660-7)
Supplement: Supplementary file 2 — Supplementary information [file 42003_2021_2660_MOESM2_ESM.pdf]

Supplementary information

The predatory soil bacterium *Lysobacter* reprograms quorum sensing system to regulate antifungal antibiotic production in a cyclic-di-GMP-independent manner

Kaihuai Li[1, 2], Gaoge Xu[1], Bo Wang[1], Guichun Wu[1], Rongxian Hou[1, 2], Fengquan Liu[1, 2, *]

[1]Institute of Plant Protection, Jiangsu Academy of Agricultural Sciences, Jiangsu Key Laboratory for Food Quality and Safety-State Key Laboratory Cultivation Base, Ministry of Science and Technology, Nanjing 210014, China

[2]College of Plant Protection, Nanjing Agricultural University, Nanjing 210095, China

Supplementary Figure 1

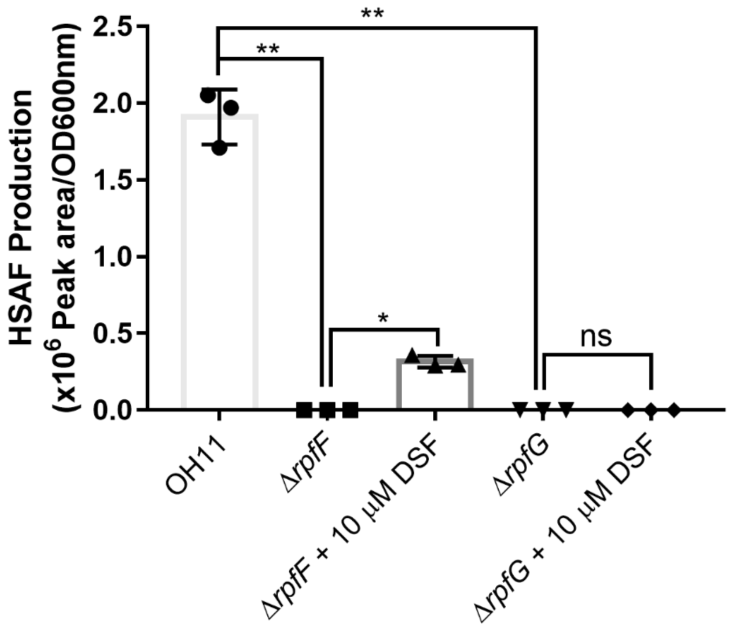

Supplementary Figure 1. DSF type-based QS systems are critical for regulating the synthesis of HSAF in *L. enzymogenes*.

Supplementary Figure 2

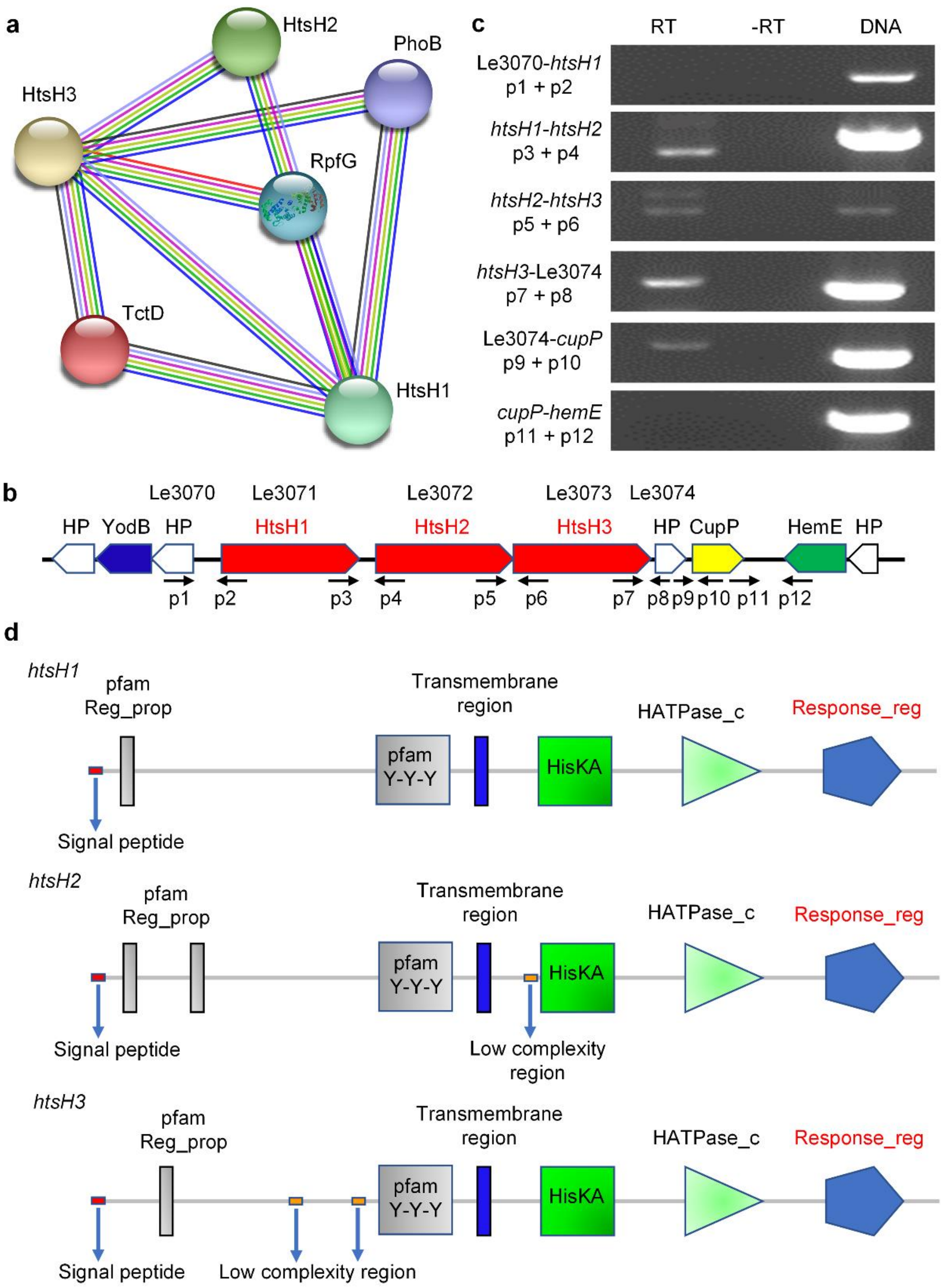

Supplementary Figure 2. *htsH1*, *htsH2* and *htsH3* are located in an operon. (a) Bioinformatics predictions that RpfG may interact with HtsH1, HtsH2 and HtsH3. **(b)** Genomic localization of *htsH1*, *htsH2* and *htsH3*. Arrows indicate open reading frame regions of the genes and their

transcriptional directions. Gene names are listed above, and primers used to verify operon structures by RT-PCR are indicated below. The primers used are listed in Supplementary Table 2. HP: hypothetical protein; YodB: cytochrome B561 (yodB); HP: hypothetical protein; HtsH1: Hybrid two-component systems protein; HtsH2: Hybrid two-component systems protein; HtsH3: Hybrid two-component systems protein; HP: hypothetical protein; CupP: Cupin 2 conserved barrel domain protein; HemE: uroporphyrinogen decarboxylase (hemE); HP: hypothetical protein. **(c)** Verification of operon organization by RT-PCR. The cDNA was reverse-transcribed with random primers using total RNA from *L. enzymogenes* grown in 10% TSB medium at 28°C until the OD600 reached 1.0. PCR fragment lengths are listed on the right. RT represents amplification using cDNA transcribed from RNA as template; -RT represents the negative control, in which reverse transcriptase was absent during cDNA synthesis; DNA represents the positive control using DNA as the PCR template. **(d)** Bioinformatics analyses of the domain organization of HtsH1, HtsH2 and HtsH3 that belong to HyTCS.

Supplementary Figure 3

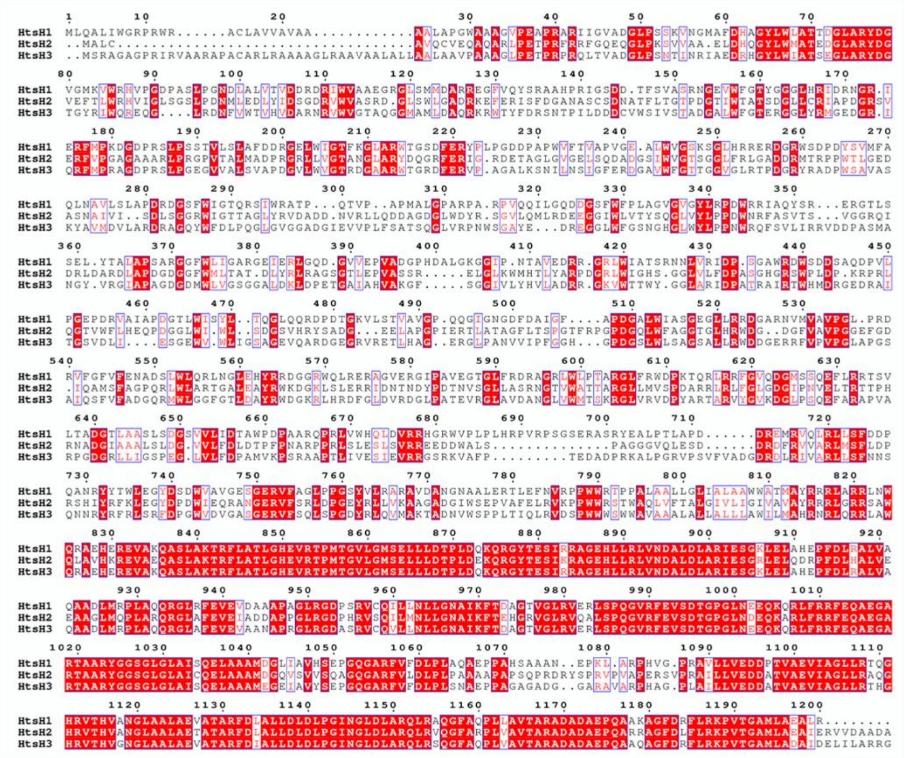

Supplementary Figure 3. Identification and sequence characterization of HtsH1, HtsH2 and HtsH3 in *L. enzymogenes*. Alignment of *L. enzymogenes* HtsH1, HtsH2 and HtsH3. The alignment was performed with Clustal W based on identical residues.

Supplementary Figure 4

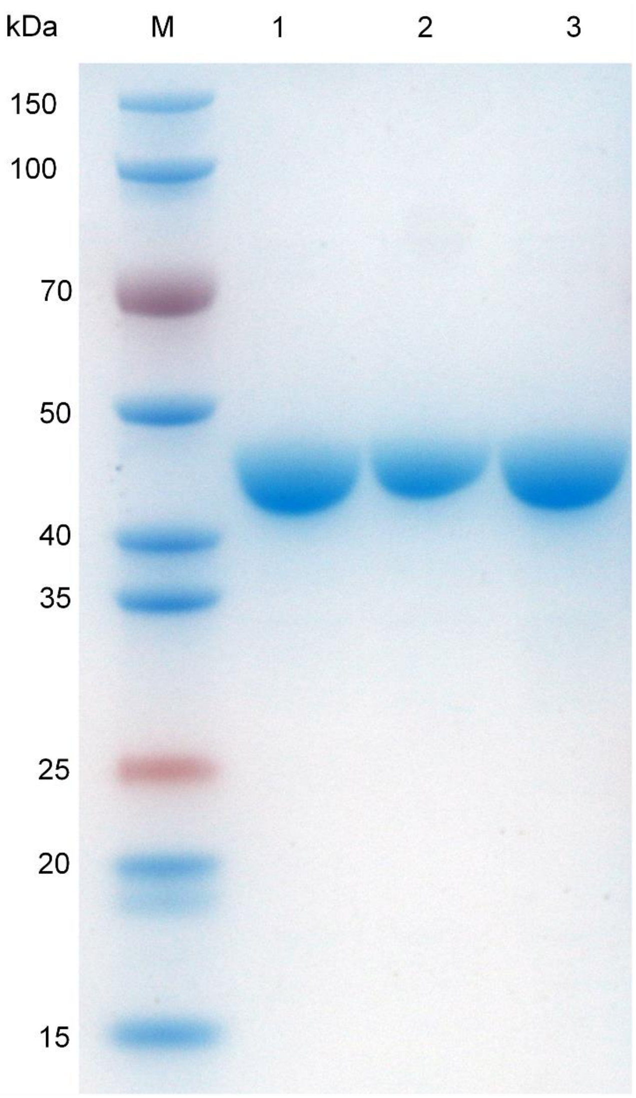

Supplementary Figure 4. The purified cytoplasmic fragments of HtsH1, HtsH2 and HtsH3 were analysed by 12% SDS-PAGE. Lane M, molecular mass markers; lane 1, HtsH1C-Flag-His protein; lane 2, HtsH2C-HA-His protein; lane 3, HtsH3C-Myc-His protein.

Supplementary Figure 5

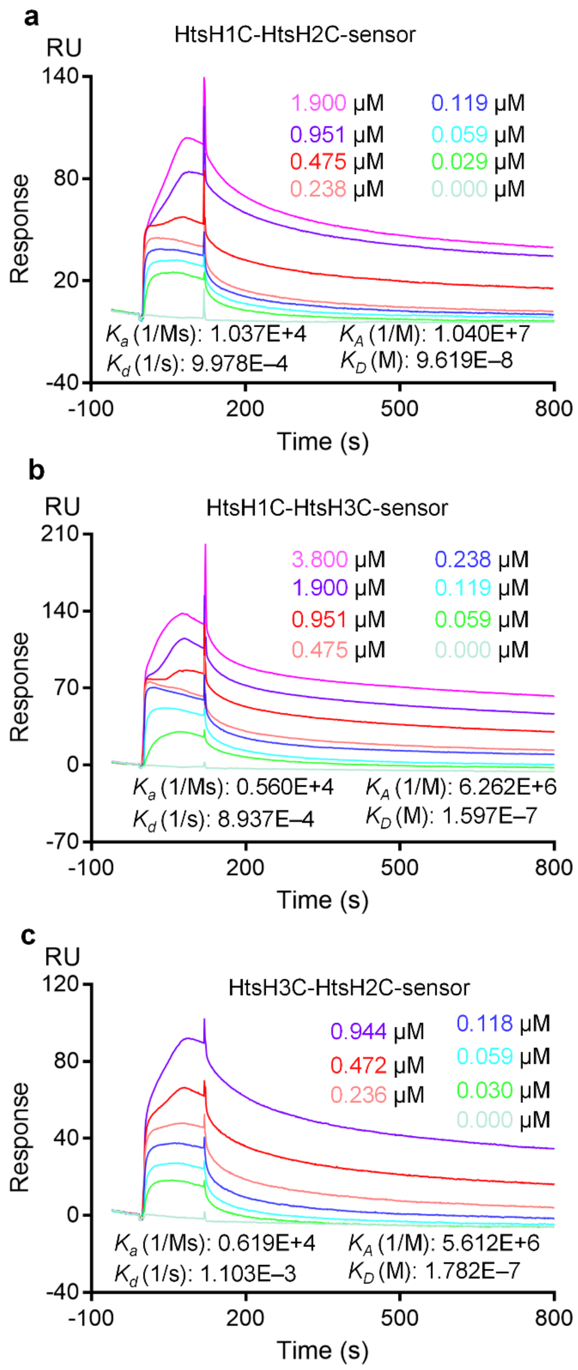

Supplementary Figure 5. HtsH1, HtsH2 and HtsH3 exist as a complex in *L. enzymogenes*. (a) SPR showing that HtsH1C-Flag-His forms a complex with HtsH2C-HA-His with K_D = 0.09619 nM. **(b)** SPR showing that HtsH1C-Flag-His forms a complex with HtsH3C-Myc-His with K_D = 0.1597 nM. **(c)** SPR showing that HtsH2C-HA-His forms a complex with HtsH3C-Myc-His with K_D = 0.1782 nM.

Supplementary Figure 6

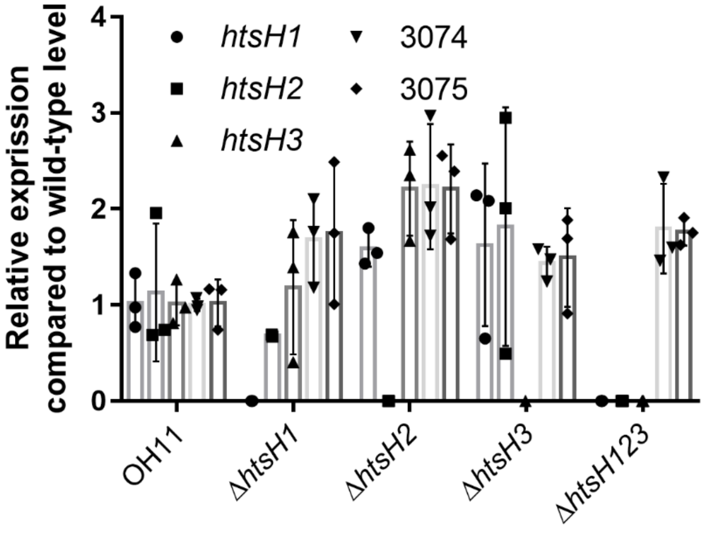

Supplementary Figure 6. The genes downstream of the *htsHs* gene deletion mutants were expressed.

Supplementary Figure 7

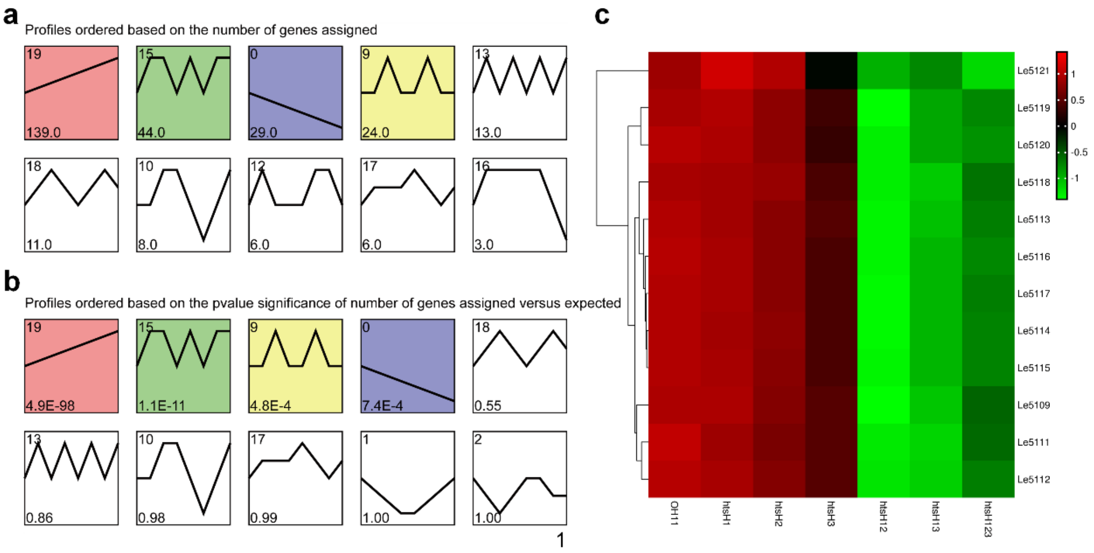

Supplementary Figure 7. Transcriptional analysis of HSAF biosynthesis genes in *L. enzymogenes*. (a-b) Trend analysis of differential gene expression in the *htsHs* mutants (Δ*htsH1*, Δ*htsH2*, Δ*htsH3*, Δ*htsH12*, Δ*htsH13*, Δ*htsH23* and Δ*htsH123*). **(c)** Hierarchical cluster analysis applied to the 12 DEGs that are significant with a p value < 0.05 in the HSAF biosynthesis gene cluster in different mutant backgrounds.

Supplementary Figure 8

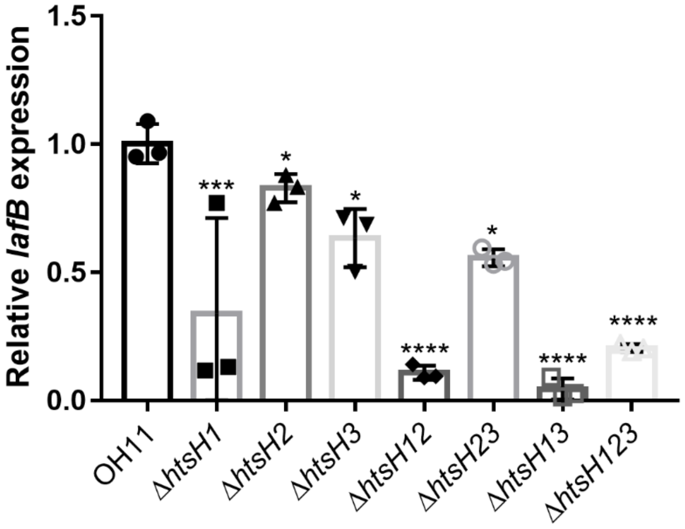

Supplementary Figure 8. qRT-PCR analyses of lafB mRNA in the wild type and htsHs mutants, grown in 10% TSB. Error bars, means ± standard deviations (n = 3). ∗ P < 0.05, ** P < 0.01, *** P < 0.001, **** P < 0.0001, assessed by one-way ANOVA. All experiments were repeated three times with similar results.

Supplementary Figure 9

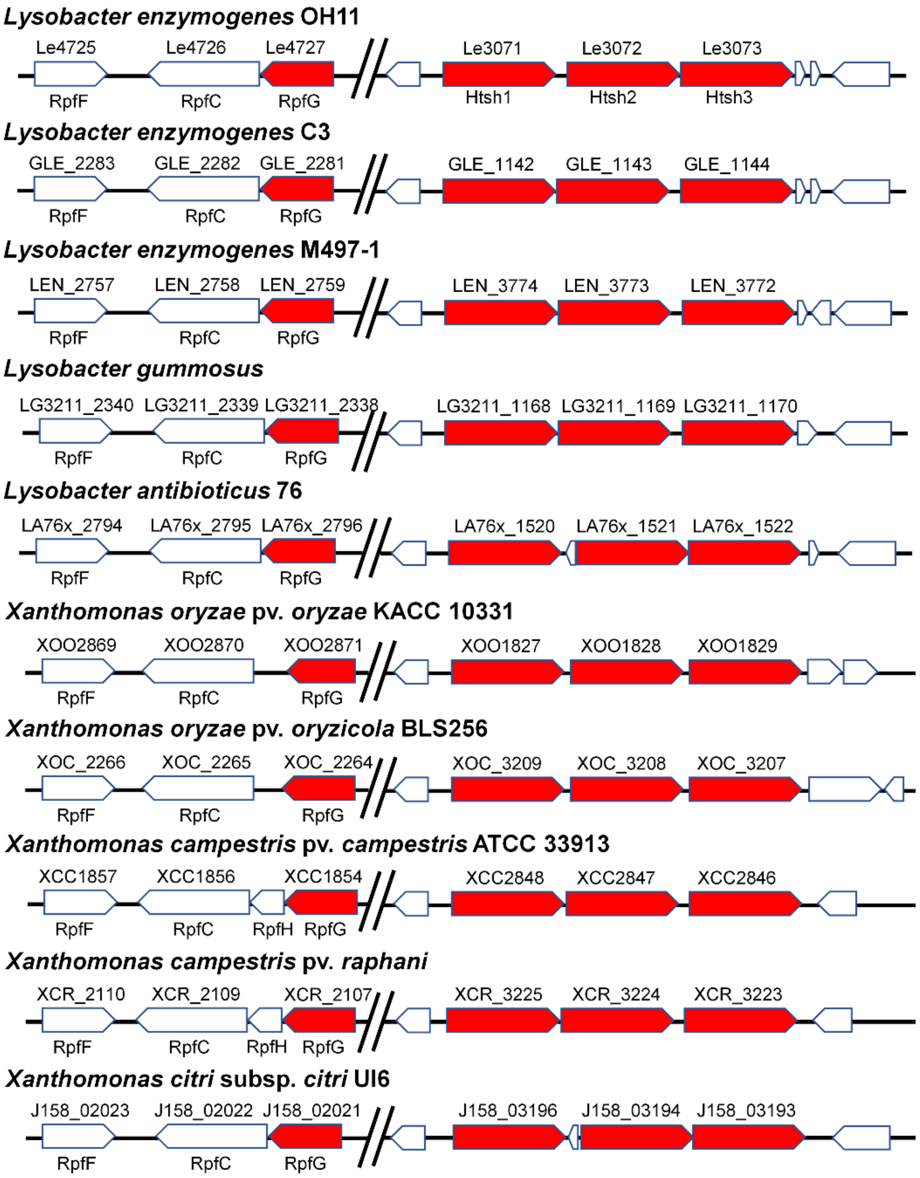

Supplementary Figure 9. Conservation of the key genes for RpfG, and HtsH1, HtsH2 and HtsH3-dependent regulatory patterns in the genomes of different bacteria. Genomic organization of the genes and homology analysis of the products. All sequences were retrieved from NCBI Microbial Genome Resources. All amino acid sequences were downloaded from the microbial genome sequence database of NCBI. Position-specific Iterated BLAST (PSI-BLAST) was used for homology analysis.

Supplementary Figure 10. Uncropped images of Coomassie-stained gels.

Fig. 1a

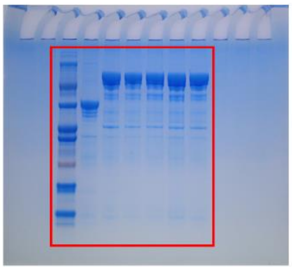

Fig. S4

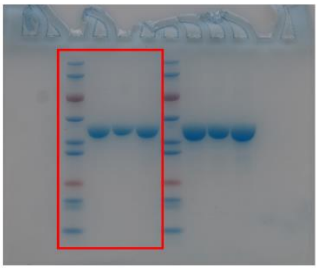

Supplementary Figure 11. Uncropped images of immunoblots.

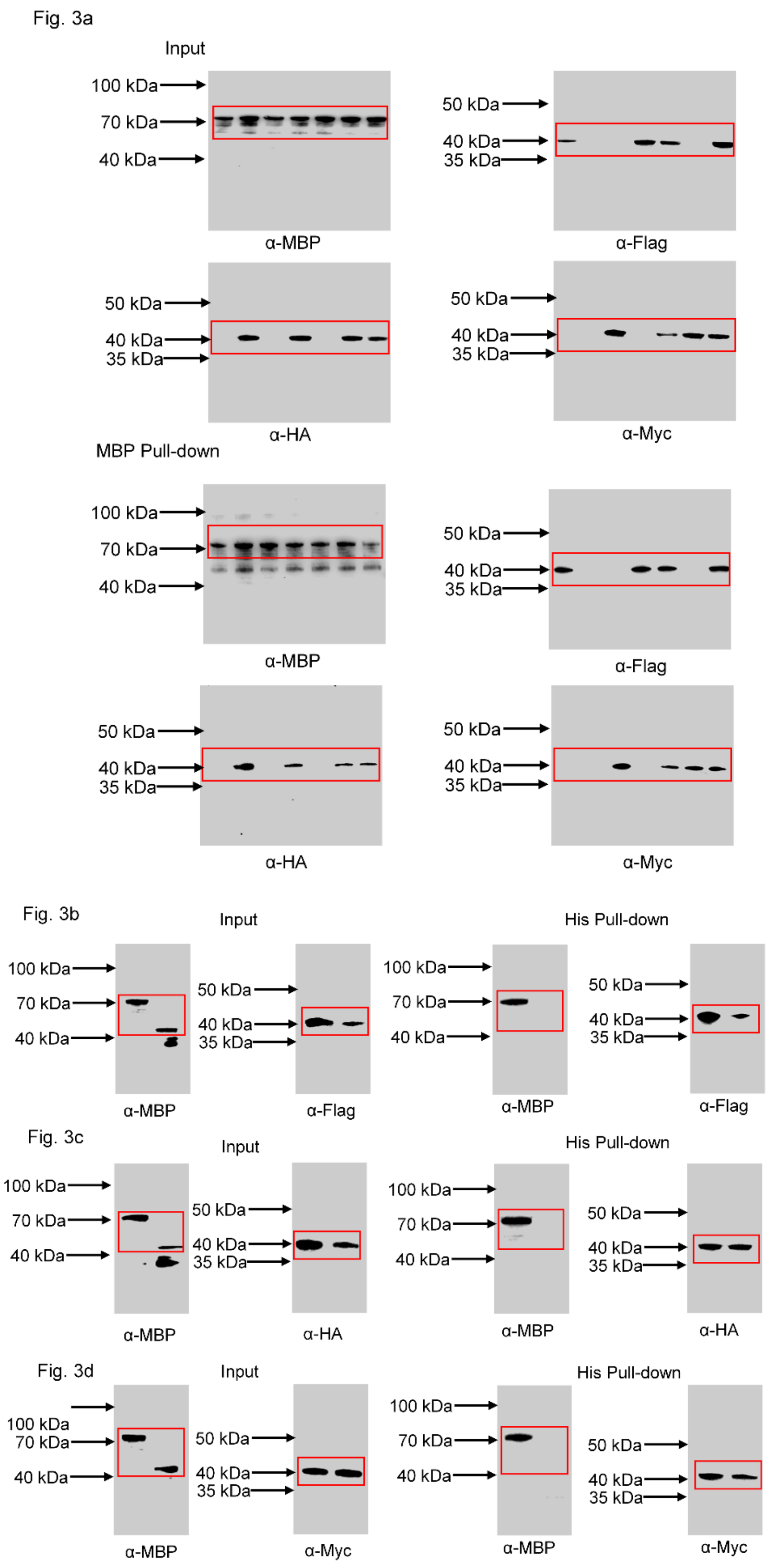

Supplementary Figure 12. Uncropped images of Mn^{2+}-Phos-tag SDS-PAGE immunoblots and EMSA.

Fig. 6b

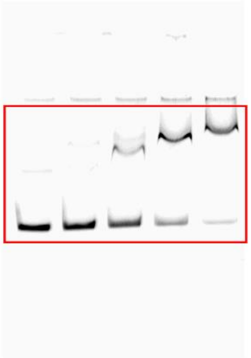

Fig. 6c

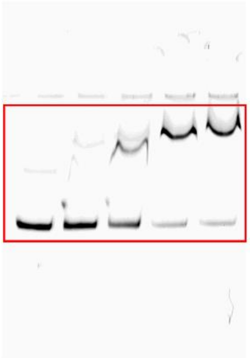

Fig. 6d

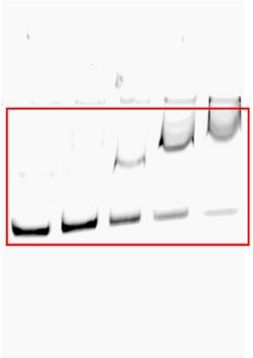

Fig. 7a

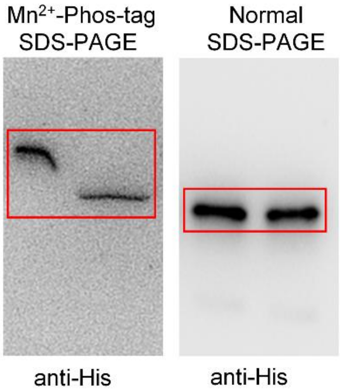

Fig. 7b

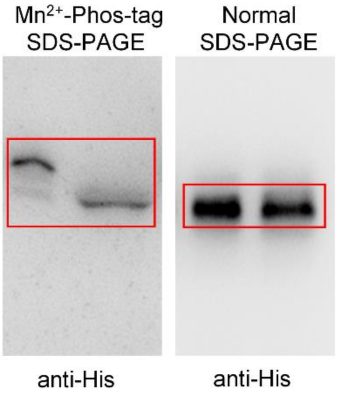

Fig. 7c

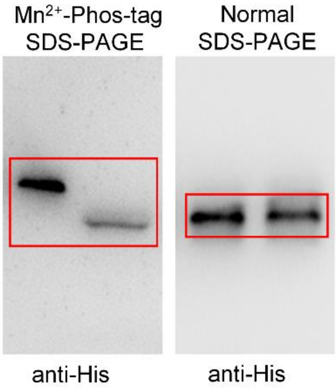

Fig. 7d

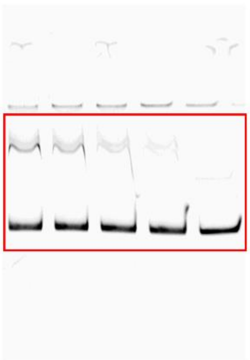

Fig. 7e

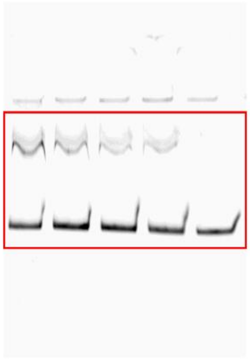

Fig. 7f

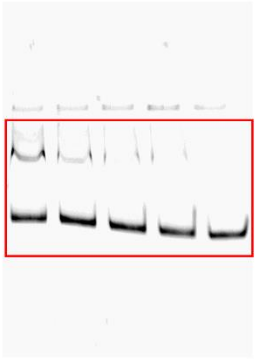

Supplementary Table 1. Bacterial strains and plasmids used in this study.

Strains	Relevant characteristics	References
E. coli		
**BL21(DE3)**	F^- *dcm omp T hsdS*($r_B^- m_B^-$) *gal* (λDE3)	Lab collection
**DH5α**	F^- *deoR endA1 gyrA96 hsdR17*($r_K^- m_K^+$) *recA1 relA1 supE*44 *thi-1* Δ(*lacZYA-argF*)*U*169(φ80*lacZ*ΔM15)	Lab collection
**XL1-Blue MRF′ kan**	Δ(*mcrA*)183, Δ(*mcrCB-hsdSMR-mrr*)173, *endA1*, *supE44*, *thi-1*, *recA1 gyrA96*, *relA1*, *lac*, [F′ *proAB lacI*^{q}ZΔ*M15* Tn5 (Km^R)]	[1]
L. enzymogenes		
**OH11**	Kan^R, wild-type strain	Lab stock
**Δ*rpfG***	Kan^R, the *rpfG* in-frame deletion mutant of strain OH11	[2]
**OH11/pBBR**	Kan^R, Gm^R, the wild-type strain harboring the plasmid pBBR1MCS5.	This study
**Δ*rpfG*/pBBR**	Kan^R, Gm^R, the *rpfG* in-frame deletion mutant harboring the plasmid pBBR1MCS5.	This study
**Δ*rpfG*/*rpfG***	Kan^R, Gm^R, the *rpfG* in-frame deletion mutant harboring the *rpfG* expression plasmid pBBR1-*rpfG*.	This study
**Δ*rpfG*/*rpfG* H190A**	Kan^R, Gm^R, the *rpfG* in-frame deletion mutant harboring the *rpfG* expression plasmid pBBR1-*rpfG* H190A.	This study
**Δ*rpfG*/*rpfG* D191A**	Kan^R, Gm^R, the *rpfG* in-frame deletion mutant harboring the *rpfG* expression plasmid pBBR1-*rpfG* D191A.	This study
**Δ*rpfG*/*rpfG* G253A**	Kan^R, Gm^R, the *rpfG* in-frame deletion mutant harboring the *rpfG* expression plasmid pBBR1-*rpfG* G253A.	This study
**Δ*rpfG*/*rpfG* Y254A**	Kan^R, Gm^R, the *rpfG* in-frame deletion mutant harboring the *rpfG* expression plasmid pBBR1-*rpfG* Y254A.	This study
**Δ*rpfG*/*rpfG* P255A**	Kan^R, Gm^R, the *rpfG* in-frame deletion mutant harboring the *rpfG* expression plasmid pBBR1-*rpfG* P255A.	This study

**Δ*htsH1***	Kan^R, the Le *htsH1* in-frame deletion mutant of strain OH11	This study
**Δ*htsH2***	Kan^R, the Le *htsH2* in-frame deletion mutant of strain OH11	This study
**Δ*htsH3***	Kan^R, the Le *htsH3* in-frame deletion mutant of strain OH11	This study
**Δ*htsH12***	Kan^R, Gm^R, the *htsH1* and *htsH2* in-frame deletion mutant of strain OH11.	This study
**Δ*htsH13***	Kan^R, Gm^R, the *htsH1* and *htsH3* in-frame deletion mutant of strain OH11.	This study
**Δ*htsH23***	Kan^R, Gm^R, the *htsH2* and *htsH3* in-frame deletion mutant of strain OH11.	This study
**Δ*htsH123***	Kan^R, Gm^R, the *htsH1*, *htsH2* and *htsH3* in-frame deletion mutant of strain OH11.	This study
**Δ*htsH1*/*H1***	Kan^R, Gm^R, the *htsH1* in-frame deletion mutant harboring the *htsH1* expression plasmid pBBR1-*htsH1*.	This study
**Δ*htsH2*/*H2***	Kan^R, Gm^R, the *htsH2* in-frame deletion mutant harboring the *htsH2* expression plasmid pBBR1-*htsH2*.	This study
**Δ*htsH3*/*H3***	Kan^R, Gm^R, the *htsH3* in-frame deletion mutant harboring the *htsH3* expression plasmid pBBR1-*htsH3*.	This study
**Δ*htsH12*/*H12***	Kan^R, Gm^R, the *htsH1* and *htsH2* double mutant strain harboring the *htsH1* and *htsH2* expression plasmid pBBR1-*htsH12*.	This study
**Δ*htsH13*/*H13***	Kan^R, Gm^R, the *htsH1* and *htsH3* double mutant strain harboring the *htsH1* and *htsH3* expression plasmid pBBR1-*htsH13*.	This study
**Δ*htsH23*/*H23***	Kan^R, Gm^R, the *htsH2* and *htsH3* double mutant strain harboring the *htsH2* and *htsH3* expression plasmid pBBR1-*htsH23*.	This study

**Δ*htsH123/H123***	Kan^R, Gm^R, the *htsH1*, *htsH2* and *htsH3* triple mutant strain harboring the *htsH1*, *htsH2* and *htsH3* expression plasmid pBBR1-*htsH123*.	This study
**Plasmids**		
**pMAL-p2x**	Amp^R; vector for expression MBP-tag fusion protein	Lab collection
**pMAL-*rpfG***	Amp^R; Le *rpfG* in pMAL-p2x	This study
**pMAL-*rpfG* H190A**	Amp^R; Le *rpfG* H190A in pMAL-p2x	This study
**pMAL-*rpfG* D191A**	Amp^R; Le *rpfG* D191A in pMAL-p2x	This study
**pMAL-*rpfG* G253A**	Amp^R; Le *rpfG* G253A in pMAL-p2x	This study
**pMAL-*rpfG* P255A**	Amp^R; Le *rpfG* P255A in pMAL-p2x	This study
**pET28(b)**	Km^R, T7 promoter-based vector expression His-tag fusion protein	Lab collection
**pET-HtsH1C**	Km^R, the cytoplasmic fragment of *htsH1* in pET-28b	This study
**pET-HtsH2C**	Km^R, the cytoplasmic fragment of *htsH2* in pET-28b	This study
**pET-HtsH3C**	Km^R, the cytoplasmic fragment of *htsH3* in pET-28b	This study
**pEX18GM**	Gm^R, *sacB*-based gene replacement vector	[3]
**pEX18-Δ*htsH1***	Gm^R, the *htsH1* in-frame deletion fragment inserted to pEX18GM vector between *Hin*dIII/*Xba* l sites	This study
**pEX18-Δ*htsH2***	Gm^R, the *htsH2* in-frame deletion fragment inserted to pEX18GM vector between *Hin*dIII/*Xba* l sites	This study
**pEX18-Δ*htsH3***	Gm^R, the *htsH3* in-frame deletion fragment inserted to pEX18GM vector between *Hin*dIII/*Xba* l sites	This study
**pEX18-Δ*htsH12***	Gm^R, the *htsH1* and *htsH2* in-frame deletion fragment inserted to pEX18GM vector between *Hin*dIII/*Xba* l sites	This study
**pEX18-Δ*htsH23***	Gm^R, the *htsH2* and *htsH3* in-frame deletion fragment inserted to pEX18GM vector between *Hin*dIII/*Xba* l sites	This study
**pEX18-Δ*htsH123***	Gm^R, the *htsH1*, *htsH2* and *htsH3* in-frame deletion fragment inserted to pEX18GM vector between *Hin*dIII/*Xba* l sites	This study

**pBBR1MCS5**	Gm^R, Broad host range cloning vector.	[4]
**pBBR1-*rpfG***	Gm^R, the *rpfG* in pBBR1MCS5	This study
**pBBR1-*rpfG* H190A**	Gm^R, the *rpfG* H190A in pBBR1MCS5	This study
**pBBR1-*rpfG* D191A**	Gm^R, the *rpfG* D191A in pBBR1MCS5	This study
**pBBR1-*rpfG* G253A**	Gm^R, the *rpfG* G253A in pBBR1MCS5	This study
**pBBR1-*rpfG* Y254A**	Gm^R, the *rpfG* Y254A in pBBR1MCS5	This study
**pBBR1-*rpfG* P255A**	Gm^R, the *rpfG* P255A in pBBR1MCS5	This study
**pBBR1-*htsH1***	Gm^R, the *htsH1* in pBBR1MCS5	This study
**pBBR1-*htsH2***	Gm^R, the *htsH2* in pBBR1MCS5	This study
**pBBR1-*htsH3***	Gm^R, the *htsH3* in pBBR1MCS5	This study
**pBBR1-*htsH12***	Gm^R, the *htsH1* and *htsH2* in pBBR1MCS5	This study
**pBBR1-*htsH13***	Gm^R, the *htsH1* and *htsH3* in pBBR1MCS5	This study
**pBBR1-*htsH23***	Gm^R, the *htsH2* and *htsH3* in pBBR1MCS5	This study
**pBBR1-*htsH123***	Gm^R, the *htsH1*, *htsH2* and *htsH3* in pBBR1MCS5	This study
**pTRG**	Tet^R, Plasmid used for protein expression in bacterial one-hybrid assay	[1]
**pTRG-HtsH1C**	Tet^R, the cytoplasmic fragment of *htsH1* in pTRG	This study
**pTRG-HtsH2C**	Tet^R, the cytoplasmic fragment of *htsH2* in pTRG	This study
**pTRG-HtsH3C**	Tet^R, the cytoplasmic fragment of *htsH3* in pTRG	This study
**pBXcmT**	Cm^R, Plasmid used for DNA cloning in bacterial one-hybridization assay	[5]
**pBXcmT-p*lafB***	Cm^R, the *lafB* promoter region in pBXcmT	This study

Supplementary Table 2. Sequences of the PCR primers used in this work.

Primer name	Primer sequence (5' to 3')	Digestion sites [a]
**For deletion**		
pEX-3071 P1	acccAAGCTTcgattgagagacatggcagc	*Hin*dIII
pEX-3071 P2	aattGGTACCcatgccgttgaccttgctc	*Kpn*I
pEX-3071 P3	aattGGTACCctgcgggccttgttcgag	*Kpn*I
pEX-3071 P4	ctagTCTAGAggtcttcgagcatgttgtcc	*Xba* I
pEX-3072 P1	acccAAGCTTctgcgggccttgttcgag	*Hin*dIII
pEX-3072 P2	aattGGTACCggtcttcgagcatgttgtcc	*Kpn*I
pEX-3072 P3	aattGGTACCtgttcctgcgcaagccggtc	*Kpn*I
pEX-3072 P4	ctagTCTAGAtgttgctgcggtcgaaatag	*Xba* I
pEX-3073 P1	acccAAGCTTgtcacccatgtcgccaac	*Hin*dIII
pEX-3073 P2	aattGGTACCcgatgcggttgatggtgttc	*Kpn*I
pEX-3073 P3	aattGGTACCgcttcgaccgtttcctgc	*Kpn*I
pEX-3073 P4	ctagTCTAGAcatacaacgtcggctggatc	*Xba* I
**For in trans expression**		
pBBR1-*htsH1* P1	atccAAGCTTtaggtgccgggatcggactg	*Hin*dIII
pBBR1-*htsH1* P1	tgcTCTAGAccttggcgcttgtgggag	*Xba* I
pBBR1-*htsH2* P1	acccAAGCTTctcccacaagcgccaagg	*Hin*dIII
pBBR1-*htsH2* P1	tgcTCTAGAcgatgcggttgatggtgttc	*Xba* I
pBBR1-*htsH3* P1	acccAAGCTTatcttgctggtggaggacga	*Hin*dIII
pBBR1-*htsH3* P1	tgcTCTAGAatgaggtttgcgtcgtaagc	*Xba* I
pBBR1-*rpfG* P1	acccAAGCTTctgctgttcccgtacatgc	*Hin*dIII
pBBR1-*rpfG* P2	atcgGGATCCaccggaaccagttcaacag	*Bam*HI
**For site-directed mutant**		
Le *rpfG* H190A P1	agatggccgcgccgctgGCAgacatcggcaagatcgc	
Le *rpfG* H190A P2	gcgatcttgccgatgtcTGCcagcggcgcggccatct	
Le *rpfG* D191A P1	tggccgcgccgctgcacGCAatcggcaagatcgccat	

Le *rpfG* D191A P2	atggcgatcttgccgatTGCgtgcagcggcgcggcca	
Le *rpfG* G253A P1	agcgctacgacggctcgGCAtatcccgacgggctggtc	
Le *rpfG* G253A P2	gaccagcccgtcgggataTGCcgagccgtcgtagcgct	
Le *rpfG* Y254A P1	gctacgacggctcgggcGCAcccgacgggctggtcggc	
Le *rpfG* Y254A P2	gccgaccagcccgtcgggTGCgcccgagccgtcgtagc	
Le *rpfG* P255A P1	acgacggctcgggctatGCAgacgggctggtcggcgag	
Le *rpfG* P255A P2	ctcgccgaccagcccgtcTGCatagcccgagccgtcgt	
**For protein expression**		
htsH1-flag P1	gaattcCATATGgcgaagacccggttcctgg	*Nde* I
htsH1-flag P2	aattGGATCCtcaTTTGTCGTCGTCGTCTTT GTAGTCggcctcgcgctcgaacaag	*Bam*HI
htsH2-HA P1	gaattcCATATGgccaagacccggttcctgg	*Nde* I
htsH2-HA P2	aattGGATCCtcaAGCGTAGTCTGGGACGT CGTATGGGTAtggcgcgtccgccgcgtcg	*Bam*HI
htsH3-Myc P1	gaattcCATATGgcgaagacgcggttcctgg	*Nde* I
htsH3-Myc P2	accgGAATTCtcaCAGATCCTCTTCTGAGA TGAGTTTTTGTTCcccttcgccgcggcgcgcga	*Eco*RI
MBP-*rpfG* P1	atcgGGATCCatgctgcgccacatcatcga	*Bam*HI
MBP-*rpfG* P2	atccAAGCTTaccggaaccagttcaacag	*Hin*dIII
**For Bacterial one-hybrid assays and EMSA**		
pTRG-3071C P1	aattGGATCCgaagtggcgaagcaggcctc	*Bam*HI
pTRG-3071C P2	aattCTCGAGccttggcgcttgtgggag	*Xho* I
pTRG-3072C P1	atcgGGATCCctggccaagacccggttcct	*Bam*HI
pTRG-3072C P2	atcgCTCGAGcgatgcggttgatggtgttc	*Xho* I
pTRG-3073C P1	accgGAATTCgagcgcgaagtggcgaagca	*Eco*RI
pTRG-3073C P2	atcgCTCGAGatgaggtttgcgtcgtaagc	*Xho* I
pBX-pHSAF P1	ccgCTCGAGctgcatctgggtgactcctg	*Xho*I
pBX-pHSAF P2	tgcTCTAGAagtgccgcagaaaatccag	*Xba*I
EMSA-pHSAF P1	acgcgctcgggtaattaacg	

EMSA-pHSAF P2	gtttcgatgatgagaccgcc
**For cotranscript identification**	
p1	taggtgccgggatcggactg
p2	catgccgttgaccttgctc
p3	gcttcgaccgcttcctgc
p4	acagggtgaactcgacgc
p5	ggttcgacctgttcctgc
p6	cgatgcggttgatggtgttc
p7	gcttcgaccgtttcctgc
p8	atgaggtttgcgtcgtaagc
p9	gcttacgacgcaaacctcat
p10	catacaacgtcggctggatc
p11	gatccagccgacgttgtatg
p12	aagggcatgtgttcaacctc
**For RT-qPCR**	
RT-16s rRNA F	acggtcgcaagactgaaact
RT-16s rRNA R	aaggcaccaatccatctctg
RT-*lafB* F	gcatccacaccgaactgaag
RT-*lafB* R	gttgagctgcttgaggaacc
RT-*htsH1* F	catctgttgcgcttggtcaa
RT-*htsH1* R	gagcaggttcagcaggatct
RT-*htsH2* F	gcctcaacgacgaacagaag
RT-*htsH2* R	ctgaccacgctgacctgt
RT-*htsH3* F	catctgttgcggttggtcaa
RT-*htsH3* R	gagcaggttcagcaagacct
RT-3074 F	acttcgcttacgacgcaaac
RT-3074 R	atggcggtcaagcaaacgc
RT-3075 F	cttcgaagcgctgttgacc
RT-3075 R	gcaggaacaggtaatcgcc

Supplementary References

1 Guo, M., Feng, H., Zhang, J., Wang, W., Wang, Y., Li, Y., Gao, C., Chen, H., Feng, Y. & He, Z. G. Dissecting transcription regulatory pathways through a new bacterial one-hybrid reporter system. *Genome Res* **19**, 1301-1308 (2009).

2 Xu, G., Han, S., Huo, C., Chin, K. H., Chou, S. H., Gomelsky, M., Qian, G. & Liu, F. Signaling specificity in the c-di-GMP-dependent network regulating antibiotic synthesis in *Lysobacter*. *Nucleic Acids Res* **46**, 9276-9288 (2018).

3 Hoang, T. T., Karkhoff-Schweizer, R. R., Kutchma, A. J. & Schweizer, H. P. A broad-host-range Flp-FRT recombination system for site-specific excision of chromosomally-located DNA sequences: application for isolation of unmarked *Pseudomonas aeruginosa* mutants. *Gene* **212**, 77-86 (1998).

4 Kovach, M. E., Elzer, P. H., Hill, D. S., Robertson, G. T., Farris, M. A., Roop, R. M., 2nd & Peterson, K. M. Four new derivatives of the broad-host-range cloning vector pBBR1MCS, carrying different antibiotic-resistance cassettes. *Gene* **166**, 175-176 (1995).

5 Xu, H., Chen, H., Shen, Y., Du, L., Chou, S. H., Liu, H., Qian, G. & Liu, F. Direct regulation of extracellular chitinase production by the transcription factor LeClp in *Lysobacter enzymogenes* OH11. *Phytopathology* **106**, 971-977 (2016).
