# Supplementary material for: The predatory soil bacterium Lysobacter reprograms quorum sensing system to regulate antifungal antibiotic production in a cyclic-di-GMP-independent manner
Source: Commun Biol. 2021 Sep 24;4:1131. doi: 10.1038/s42003-021-02660-7 (PMC8463545; doi:10.1038/s42003-021-02660-7)
Supplement: Supplementary file 3 — Description of Additional Supplementary Files [file 42003_2021_2660_MOESM3_ESM.pdf]

Description of additional Supplementary Files

File name: Supplementary Data 1
Description: List of genes differentially expressed in the *htsH1*, *htsH2*, and *htsH3* mutants compared to the wild-type strain.

File name: Supplementary Data 2
Description: Source data of main figures.
