# Supplementary material for: The predatory soil bacterium Lysobacter reprograms quorum sensing system to regulate antifungal antibiotic production in a cyclic-di-GMP-independent manner
Source: Commun Biol. 2021 Sep 24;4:1131. doi: 10.1038/s42003-021-02660-7 (PMC8463545; doi:10.1038/s42003-021-02660-7)
Supplement: Supplementary file 4 — Supplementary Data 1 [file 42003_2021_2660_MOESM4_ESM.docx]

**Supplementary Data 1.** List of genes differentially expressed in the *htsH1*, *htsH2*, and *htsH3* mutants compared to the wild-type strain.

| Gene name | log2 fold change (mutant strian / wild-type) | | | | | | | | Gene description |
| --- | --- | --- | --- | --- | --- | --- | --- | --- | --- |
|  | OH11 | Δ*htsH1* | Δ*htsH2* | Δ*htsH3* | Δ*htsH12* | Δ*htsH23* | Δ*htsH13* | Δ*htsH123* |  |
| Le0029 | 0 | 0.71 | 0.88 | -0.18 | 0.3 | 0.82 | 1.25 | 1.2 | hypothetical protein |
| Le0030 | 0 | 0.44 | 0.6 | 0.83 | 0.65 | 0.69 | 0.52 | 1.06 | hypothetical protein |
| Le0055 | 0 | 0.46 | 0.84 | 0.28 | 0.83 | 0.91 | 1.2 | 1.18 | hypothetical protein |
| Le0059 | 0 | 0.42 | 0.47 | 0.46 | 0.16 | 0.4 | 0.33 | 1.3 | AsnC family transcriptional regulator |
| Le0060 | 0 | 1.12 | 1.71 | 1.64 | 0.14 | 1.66 | 1.34 | 2.8 | hypothetical protein |
| Le0082 | 0 | 0.68 | 0.57 | 0.61 | 0.79 | 0.72 | 0.82 | 1.06 | putative transmembrane GGDEF transcriptional regulatory protein |
| Le0161 | 0 | 0.91 | 0.57 | 0.46 | 0.48 | 0.78 | 0.9 | 1.05 | aldehyde-activating protein |
| Le0183 | 0 | 0.9 | 0.9 | 1.58 | 1.94 | 1.82 | 1.5 | 2.76 | putative methyltransferase |
| Le0241 | 0 | 0.63 | -0.02 | -0.52 | 0.3 | 0.52 | 0.95 | 1.1 | type IV pilus modification protein PilV |
| Le0277 | 0 | 0.63 | -0.04 | 0.4 | 0.59 | 0.96 | 1.28 | 1.42 | hypothetical protein |
| Le0305 | 0 | 1.17 | 0.13 | -0.37 | -1.97 | 1.09 | -1.72 | -1.29 | hypothetical protein |
| Le0306 | 0 | 0.06 | -0.92 | -1.25 | -2.16 | -0.41 | -2.42 | -2.91 | C4-dicarboxylate ABC transporter |
| Le0317 | 0 | 0.57 | 0.95 | 0.71 | 0.25 | 0.87 | -0.72 | 1.92 | hypothetical protein |
| Le0320 | 0 | 0.77 | 0.21 | 0.24 | 0.52 | 0.71 | 0.85 | 1.04 | glycosyl hydrolase family 5 |
| Le0336 | 0 | 1 | 0.23 | -0.18 | 0.74 | 0.65 | 1.12 | 1.11 | hypothetical protein |
| Le0347 | 0 | 0.87 | 0.35 | 0.81 | 0.64 | 0.65 | 0.49 | 1.05 | conserved hypothetical protein |
| Le0365 | 0 | 1.27 | 0.6 | 0.91 | 0.74 | 1.19 | 0.16 | 1.4 | putative Na^+^-dependent transporter |
| Le0383 | 0 | 0.57 | 0.31 | 0.45 | 0.79 | 0.52 | 0.72 | 1.2 | hypothetical protein |
| Le0446 | 0 | 0.72 | 0.57 | 0.78 | 1.17 | 0.62 | 0.76 | 1.29 | outer membrane lipoprotein LolB |
| Le0454 | 0 | 0.8 | 0.77 | 1.15 | 1.51 | 0.52 | 1.16 | 2.12 | hypothetical protein |
| Le0493 | 0 | 0.83 | 0.61 | 0.53 | 0.63 | 0.53 | 1.02 | 1.19 | hypothetical protein |
| Le0496 | 0 | 0.86 | 0.34 | 0.34 | 1.16 | -0.17 | -0.11 | 1.63 | AraC-type DNA binding HTH domain |
| Le0503 | 0 | 0.69 | 0.28 | 0.52 | 0.67 | 0.59 | 0.8 | 1.06 | RNA polymerase sigma70 factor |
| Le0533 | 0 | 0.51 | 0.46 | 0.82 | 0.14 | 0.88 | 1 | 1.03 | protein of unknown function DUF6 transmembrane |
| Le0534 | 0 | 2.15 | 0.88 | 0.56 | 1.88 | 1.78 | 2.17 | 2.08 | hypothetical protein |
| Le0592 | 0 | 1.23 | 0.75 | 0.2 | 1.08 | 0.72 | 1.37 | 1.17 | Signal transduction histidine kinase |
| Le0659 | 0 | 0.97 | 1.09 | 1.06 | 0.97 | 1.23 | 1.02 | 1.8 | hypothetical protein |
| Le0660 | 0 | 0.64 | 0.63 | 1.05 | 1.07 | 1.32 | 1.21 | 1.65 | hypothetical protein |
| Le0797 | 0 | 0.67 | 0.9 | 1.02 | 0.32 | 0.96 | 1.64 | 1.34 | CAAX amino terminal protease |
| Le0836 | 0 | 0.45 | 0.54 | 0.67 | 0.97 | 0.38 | 0.74 | 1.02 | IgA-specific serine endopeptidase |
| Le0840 | 0 | 0.93 | 0.67 | 1.04 | 1.47 | 1.25 | 1.51 | 1.36 | Acetyltransferase, GNAT family protein |
| Le0863 | 0 | 2.22 | 2.06 | 2.25 | 2.96 | 1.46 | 2.65 | 4.08 | hypothetical protein |
| Le0872 | 0 | 2.8 | 4.46 | 3.4 | 2 | 3.22 | 2.39 | 3.62 | hypothetical protein |
| Le0873 | 0 | 1.34 | -0.13 | -0.28 | 0.73 | 0.96 | 0.9 | 2.57 | hypothetical protein |
| Le0931 | 0 | -0.1 | 0.86 | -0.55 | -1.4 | 0.17 | -1.46 | -1.91 | sulfotransferase protein |
| Le0932 | 0 | 0.07 | 0.86 | -0.72 | -1.21 | 0.13 | -1.19 | -1.68 | short-chain dehydrogenase/reductase SDR |
| Le0933 | 0 | -0.18 | 0.9 | -0.6 | -1.34 | 0.06 | -1.43 | -1.75 | hypothetical protein |
| Le0934 | 0 | -0.1 | 0.97 | -0.54 | -1.4 | 0.16 | -1.1 | -1.59 | coproporphyrinogen III oxidase |
| Le0935 | 0 | -0.45 | 0.73 | -0.76 | -1.69 | -0.07 | -1.64 | -2.29 | hypothetical protein |
| Le0938 | 0 | 0.33 | 1.07 | -0.39 | -1.15 | 0.59 | -0.8 | -1.07 | uncharacterized protein |
| Le0939 | 0 | 0.31 | 1 | -0.7 | -1 | 0.13 | -0.7 | -1.18 | inner membrane transport permease |
| Le0941 | 0 | 0.34 | 0.77 | -0.37 | -1.15 | 0.07 | -1.01 | -1.07 | hypothetical protein |
| Le0962 | 0 | 0.71 | 0.29 | 0.18 | 0.79 | 0.27 | 0.95 | 1.28 | DUF11 domain-containing protein |
| Le1035 | 0 | 1.11 | 1.43 | 0.13 | 0.47 | 0.58 | 0.71 | 1.82 | acetyltransferase |
| Le1036 | 0 | 0.91 | 1.21 | 0.49 | 0.27 | 0.81 | 0.89 | 1.07 | integral membrane-like protein |
| Le1065 | 0 | 1.81 | 1.29 | 1.41 | 1.84 | 1.22 | 2.74 | 2.31 | cell envelope biogenesis protein TonB |
| Le1066 | 0 | 0.89 | 1.05 | 1.28 | 1.61 | 1.39 | 1.32 | 1.6 | hypothetical protein |
| Le1074 | 0 | 0.64 | 0.41 | 0.54 | 0.44 | 0.15 | 0.92 | 1.02 | XshC-Cox1-family protein |
| Le1100 | 0 | 0.99 | 0.95 | 0.54 | 0.98 | 0.61 | 0.96 | 1.09 | transglutaminase |
| Le1108 | 0 | 0.37 | -0.08 | 0.25 | 0.47 | 0.48 | 0.09 | 1.05 | X-Pro dipeptidase |
| Le1109 | 0 | 0.25 | -0.09 | 0.47 | 0.18 | 0.37 | 0.19 | 1.12 | aldehyde dehydrogenase (NAD+) |
| Le1114 | 0 | 0.17 | 0.01 | 0.06 | -0.13 | 0.18 | 0.06 | 1.08 | hydroxyproline-2-epimerase |
| Le1124 | 0 | 0.45 | -0.11 | 0.18 | 0.46 | 0.5 | 0.56 | 1.02 | hypothetical protein |
| Le1126 | 0 | -0.3 | -0.41 | -1.02 | -0.87 | -0.24 | -0.73 | -1.09 | flagellar biosynthesis protein FliQ |
| Le1173 | 0 | 0.34 | 0.63 | 0.49 | 0.32 | 0.54 | 1.39 | 1.02 | AsnC family transcriptional regulator |
| Le1188 | 0 | 1.09 | 0.38 | 0.13 | 0.02 | 0.62 | 0.75 | 1.33 | hypothetical protein |
| Le1213 | 0 | 1.08 | 0.04 | -0.22 | 0.19 | -0.35 | 0.32 | 1.36 | hypothetical protein |
| Le1228 | 0 | 0.33 | 0.71 | 0.49 | 0.47 | 0.73 | 0.57 | 1.09 | hypothetical protein |
| Le1250 | 0 | 0.65 | 0.16 | 0.25 | 0.09 | 0.68 | 0.51 | 1.09 | hypothetical protein |
| Le1259 | 0 | -0.08 | 0.33 | -0.93 | -1.27 | 0.05 | -0.82 | -1.23 | PAAR domain-containing protein |
| Le1296 | 0 | 2.1 | 2.33 | 1.27 | 1.19 | 1.42 | 1.79 | 2.24 | hypothetical protein |
| Le1301 | 0 | -0.09 | 0.73 | -0.85 | -1.61 | -0.21 | -1.2 | -1.7 | Aminotransferase, classes I and II |
| Le1302 | 0 | 0.02 | 0.98 | -0.47 | -1.37 | 0.2 | -1.04 | -1.59 | L-2, 4-diaminobutyrate decarboxylase |
| Le1312 | 0 | 1.05 | 0.58 | 0.05 | 0.55 | 1.36 | 0.55 | 1.06 | hypothetical protein |
| Le1330 | 0 | 1.38 | 0.69 | 1.28 | 0.61 | 1.49 | 1.6 | 1.78 | hypothetical protein |
| Le1348 | 0 | 0.74 | 1.12 | 0.34 | 0.87 | 0.45 | 0.95 | 1.41 | Putative secreted protein |
| Le1439 | 0 | 1.19 | 0.27 | 1.04 | 0.38 | 0.58 | 0.96 | 1.3 | hypothetical protein |
| Le1440 | 0 | 0.84 | 0.58 | 0.99 | 0.8 | 0.48 | 0.89 | 1.27 | hypothetical protein |
| Le1462 | 0 | 1.26 | 0.31 | 0.75 | 1.74 | 0.57 | 0.6 | 1.68 | rrf2 family protein transcriptional regulator family protein |
| Le1491 | 0 | 0.56 | 0.8 | 1.13 | 1.13 | 1.09 | 0.94 | 1.69 | hypothetical protein |
| Le1503 | 0 | 0.69 | 0.22 | 0.86 | 0.66 | 0.56 | 0.25 | 1.52 | TonB-dependent receptor |
| Le1513 | 0 | 1.14 | 0.79 | 0.52 | 0.88 | 1.66 | 1.26 | 1.97 | uroporphyrinogen-III synthase |
| Le1537 | 0 | 0.7 | 0.68 | 0.52 | 0.91 | 0.46 | 0.84 | 1.3 | two-component system regulatory protein |
| Le1544 | 0 | 1.03 | 0.53 | -0.23 | 0.43 | 0.72 | 1.26 | 1.18 | hypothetical protein |
| Le1575 | 0 | 0.75 | 0.17 | 0.63 | 0.65 | 0.38 | 0.52 | 1.15 | hypothetical protein |
| Le1581 | 0 | 0.49 | 0.47 | 0.5 | 0.67 | 0.55 | 0.73 | 1.05 | hypothetical protein |
| Le1594 | 0 | 1.2 | 0.76 | 0.18 | 1.2 | 1.1 | 1.54 | 1.33 | hypothetical protein |
| Le1597 | 0 | 0.66 | 0.49 | 0.68 | 0.38 | 0.97 | 1.11 | 1.18 | hypothetical protein |
| Le1634 | 0 | 1.52 | 1.78 | 1.66 | 1.8 | 0.59 | 1.55 | 2.16 | hypothetical protein |
| Le1675 | 0 | 1.66 | 1.97 | 1.63 | 1.75 | 1.97 | 2.61 | 3.12 | transmembrane anti-sigma factor |
| Le1677 | 0 | -0.38 | -0.22 | -0.46 | -0.09 | 0.32 | 0.4 | -3.82 | catalase |
| Le1707 | 0 | 0.66 | 0.58 | 0.18 | 0.27 | 0.83 | 0.98 | 1.09 | exodeoxyribonuclease V subunit alpha |
| Le1730 | 0 | 0.2 | 0.53 | 0.22 | 0.64 | 0.9 | 0.68 | 1.08 | AsnC family transcriptional regulator |
| Le1743 | 0 | 0.76 | 1.55 | 1.07 | 1.42 | 0.92 | 1.94 | 2.01 | two-component system sensor protein |
| Le1780 | 0 | 1.81 | 1.56 | 0.91 | 1.4 | 1.21 | 1.36 | 1.74 | SIMPL domain-containing protein |
| Le1827 | 0 | 1.18 | 1.57 | 0.99 | 1.48 | 0.5 | 1.58 | 2.12 | hypothetical protein CATMIT_01610, partial |
| Le1863 | 0 | 0.81 | 0.87 | 0.28 | 0.75 | 0.36 | 0.43 | 1.51 | hypothetical protein |
| Le1898 | 0 | 1 | 0.48 | 0.57 | 0.15 | 0.25 | 0.74 | 1.03 | hypothetical protein |
| Le1914 | 0 | 0.78 | 0.02 | 0.41 | 0.26 | 0.65 | 0.76 | 1.25 | Agmatinase |
| Le1920 | 0 | 1.26 | 0.78 | 0.35 | 1.03 | 0.89 | 0.84 | 1.19 | hypothetical protein |
| Le1926 | 0 | 0.85 | 0.18 | 0.88 | 1.04 | 2 | 1.13 | 2.38 | fimbrial protein |
| Le1967 | 0 | 0.73 | 1.38 | -0.97 | -2.05 | 0.84 | -0.29 | -1.32 | hypothetical protein |
| Le1993 | 0 | 0.44 | 0.55 | 0.53 | 0.59 | 0.65 | 1.02 | 1.42 | hypothetical protein |
| Le2014 | 0 | 2.08 | 2.14 | 1.78 | 2.16 | 1.84 | 2.42 | 2.31 | hypothetical protein |
| Le2016 | 0 | 0.73 | 0.61 | 0.36 | 0.94 | 0.65 | 1 | 1.25 | hypothetical protein |
| Le2029 | 0 | 1.62 | 1.67 | 1.81 | 1.48 | 0.09 | 2.53 | 2.7 | RDD family protein |
| Le2049 | 0 | 0.18 | 0.03 | -0.11 | 0.32 | 0.19 | 0.34 | 1.24 | multidrug ABC transporter ATP-binding protein |
| Le2056 | 0 | 0.28 | 0.46 | 0.54 | 1.01 | 0.68 | 0.86 | 1.04 | two-component system response regulator, LuxR family |
| Le2156 | 0 | 1.02 | 0.71 | 0.76 | 0.98 | 0.76 | 0.77 | 1.33 | GCN5 family acetyltransferase |
| Le2177 | 0 | 0.03 | 0.67 | 0.58 | 0.98 | -0.29 | 0.42 | 2 | MarR family transcriptional regulator, partial |
| Le2180 | 0 | 0.53 | 0.51 | 0.15 | 0.61 | 0.86 | 0.89 | 1.06 | hypothetical protein |
| Le2189 | 0 | 1.07 | 0.64 | 0.83 | 1.08 | 0.99 | 0.95 | 1.83 | hypothetical protein |
| Le2194 | 0 | 0.64 | 0.63 | 0.29 | 0.98 | 0.45 | 0.94 | 1.11 | Xaa-Pro dipeptidase, putative |
| Le2211 | 0 | 1.17 | 0.8 | 0.37 | 0.72 | 0.85 | 0.92 | 1.22 | hypothetical protein |
| Le2224 | 0 | 0.53 | 0.75 | 0.36 | 0.88 | 0.51 | 0.67 | 1.17 | protein of unknown function DUF81 |
| Le2228 | 0 | -10.93 | -4.7 | -6.02 | -10.93 | -4.43 | -4.6 | -5.6 | transcriptional regulator |
| Le2263 | 0 | 1.63 | 0.92 | 0.71 | 0.59 | 0.73 | 1.42 | 1.4 | hypothetical protein |
| Le2273 | 0 | 0.19 | 0.11 | 1.05 | 0.82 | 0.47 | 0.92 | 1.03 | transcriptional regulator |
| Le2311 | 0 | -0.16 | 0.65 | -0.36 | -1.35 | 0.26 | -0.8 | -1.14 | hypothetical protein |
| Le2326 | 0 | 0.14 | 1.46 | -0.57 | -1.88 | 0.8 | -1.24 | -1.73 | ImcF-related family protein |
| Le2327 | 0 | 0.21 | 1.52 | -0.56 | -2.1 | 1.05 | -1.53 | -2.23 | type VI secretion protein |
| Le2328 | 0 | 0 | 1.2 | -0.71 | -1.81 | 1.07 | -1.42 | -1.53 | cell envelope biogenesis protein OmpA |
| Le2338 | 0 | 0.18 | 1.32 | -0.57 | -1.43 | 0.73 | -0.71 | -1.49 | type IV secretion protein Rhs |
| Le2343 | 0 | -0.05 | 0.87 | -0.52 | -1.4 | 0.5 | -0.9 | -1.18 | hypothetical protein |
| Le2344 | 0 | 0.07 | 1.09 | -0.51 | -1.59 | 0.54 | -0.9 | -1.3 | conserved hypothetical protein |
| Le2345 | 0 | 0.27 | 1.2 | -0.45 | -1.49 | 0.68 | -0.91 | -1.48 | EvpB family type VI secretion protein |
| Le2346 | 0 | 0.24 | 1.29 | -0.42 | -2.03 | 1.05 | -1.21 | -1.93 | Hcp family protein |
| Le2348 | 0 | 0.3 | 1.63 | -0.17 | -1.73 | 0.92 | -1.17 | -1.46 | type VI secretion protein |
| Le2349 | 0 | 0.31 | 1.44 | -0.36 | -1.74 | 0.81 | -0.75 | -1.71 | type VI secretion protein |
| Le2350 | 0 | -0.11 | 1.32 | -0.43 | -2.04 | 1.01 | -1.59 | -1.66 | hypothetical protein |
| Le2351 | 0 | 0.05 | 1.56 | -0.52 | -1.96 | 0.93 | -1.16 | -1.79 | type VI secretion ATPase, ClpV1 family |
| Le2388 | 0 | 1.13 | 0.21 | 0.93 | 0.08 | 1 | 1.4 | 1.13 | hypothetical protein |
| Le2399 | 0 | -0.4 | -0.88 | -0.58 | -1.59 | -0.16 | -0.9 | -1.1 | LysM peptidoglycan-binding domain-containing protein |
| Le2400 | 0 | -0.32 | -0.68 | -0.59 | -1.71 | -0.1 | -0.93 | -1.12 | Rare lipoprotein A |
| Le2428 | 0 | 0.71 | -0.05 | 0.42 | 0.29 | 0.7 | 0.85 | 1.04 | hypothetical protein |
| Le2435 | 0 | 0.13 | 0.47 | -0.47 | -1.48 | 0.04 | -0.02 | -1.55 | conserved protein of unknown function |
| Le2449 | 0 | 1.09 | 1.12 | 0.33 | 0.72 | 1.24 | 1.21 | 1.83 | hypothetical protein |
| Le2457 | 0 | 1.11 | 0.64 | -0.08 | 1.06 | 0.76 | 0.72 | 1.2 | bacterial regulatory helix-turn-helix protein, LysR family protein 1 |
| Le2461 | 0 | -0.55 | 3.12 | -1.16 | -4.32 | 1.33 | -3.12 | -3.04 | DUF2188 domain-containing protein |
| Le2485 | 0 | 0.54 | 0.38 | 0.89 | 0.63 | 0.68 | 1.1 | 1.29 | TetR family transcriptional regulator |
| Le2492 | 0 | 0.34 | -0.13 | 0.16 | 0.5 | 0.75 | 0.75 | 1.05 | hypothetical protein |
| Le2506 | 0 | 1.36 | 1.33 | 1.2 | 1.34 | 2 | 1.68 | 1.7 | hypothetical protein |
| Le2520 | 0 | 1.28 | 0.8 | 0.97 | 0.94 | 1.13 | 1.27 | 2 | hypothetical protein |
| Le2542 | 0 | 0.39 | 0.2 | 0.43 | 0.62 | 0.53 | 1.06 | 1.2 | glyoxalase |
| Le2591 | 0 | 1.25 | 0.7 | 0.66 | 1.02 | 0.91 | 0.46 | 1.08 | dithiobiotin synthetase |
| Le2592 | 0 | 0.18 | 0.23 | -1.46 | -0.94 | 0.12 | -0.29 | -1.44 | hypothetical protein |
| Le2595 | 0 | 1.03 | 0.46 | 0.15 | 0.11 | 0.55 | 0.65 | 1.16 | bacterioferritin |
| Le2634 | 0 | 0.85 | 1.35 | 0.66 | 0.93 | 0.34 | 0.87 | 1.25 | hypothetical protein |
| Le2645 | 0 | 0.3 | 2.32 | 1.23 | 1.44 | 2.19 | 2.05 | 2.26 | hypothetical protein |
| Le2647 | 0 | 0.59 | 0.29 | 0.6 | 0.45 | 0.72 | 0.86 | 1.23 | hypothetical protein involved in tolerance to divalent cations |
| Le2715 | 0 | 0.34 | 0.23 | 0.12 | 0.73 | 0.42 | 0.68 | 1.26 | hypothetical protein |
| Le2716 | 0 | 1.03 | 0.8 | 0.89 | 1.02 | 0.74 | 1.58 | 1.84 | hypothetical protein |
| Le2727 | 0 | 0.9 | 0.8 | 0.65 | 0.93 | 0.96 | 1.12 | 1.11 | transcriptional regulator, AraC family |
| Le2732 | 0 | 1.53 | 1.75 | 1.3 | 1.86 | 0.7 | 1.98 | 2.25 | serine/threonine protein kinase |
| Le2774 | 0 | 0.19 | 0.5 | 0.41 | 0.6 | 0.45 | 0.77 | 1.79 | hypothetical protein |
| Le2799 | 0 | 0.71 | 0.27 | -0.26 | 0.64 | 0.52 | 0.87 | 1.07 | fimbrial assembly protein |
| Le2809 | 0 | 0.86 | 0.62 | 0.08 | 0.24 | 0.72 | 0.94 | 1.03 | hypothetical protein |
| Le2821 | 0 | 1.01 | 1.85 | 2.09 | 2.45 | 0.43 | 1.9 | 3.53 | hypothetical protein |
| Le2846 | 0 | 0.8 | 0.83 | 0.78 | 0.59 | 1.09 | 0.63 | 1.51 | hypothetical protein |
| Le2870 | 0 | 0.86 | 0.76 | 1.1 | 0.07 | 1.44 | 0.42 | 1.48 | hypothetical protein |
| Le2884 | 0 | 1.52 | 0.82 | 1.15 | 0.2 | 1.59 | 0.84 | 1.55 | conserved hypothetical protein |
| Le2908 | 0 | 0.43 | 0.64 | 0.61 | 0.57 | 0.61 | 0.85 | 1.21 | hypothetical protein |
| Le2911 | 0 | 1.05 | 0.7 | 0.91 | 0.83 | 0.8 | 0.96 | 1.48 | putative transcriptional acitvator, Baf family |
| Le2912 | 0 | 0.55 | 0.32 | 0.23 | 0.7 | 0.36 | 0.94 | 1.04 | sporulation protein |
| Le2936 | 0 | 1.33 | 0.74 | 1.23 | 1.41 | 0.47 | 1.13 | 1.63 | hypothetical protein |
| Le2937 | 0 | 0.31 | 0.13 | 0.51 | 0.69 | 0.25 | 0.76 | 1.01 | aminopeptidase |
| Le3071 | 0 | -13.47 | 0.69 | -0.12 | -13.47 | 0.29 | -13.47 | -13.47 | histidine kinase |
| Le3072 | 0 | 0.76 | -2.9 | 0.22 | -14.44 | -0.53 | 0.81 | -14.44 | histidine kinase |
| Le3073 | 0 | 0.74 | 0.53 | -13.96 | -1.48 | -0.59 | -8.17 | -13.96 | two component system sensor-response regulator hybrid protein |
| Le3075 | 0 | 1.76 | 0.14 | 1.37 | 1.22 | 1.84 | 1.95 | 2.25 | Cupin 2 conserved barrel domain protein |
| Le3080 | 0 | 0.96 | 0.49 | 0.92 | 0.81 | 1.06 | 1.34 | 1.24 | transcriptional regulator containing an amidase domain and an AraC-type DNA-binding HTH domain |
| Le3084 | 0 | 0.87 | 0.59 | 0.74 | 0.61 | 0.75 | 1.2 | 1.04 | D-glycerate 3-kinase |
| Le3087 | 0 | 0.71 | -0.09 | 2.01 | 1.21 | 0.81 | 1.53 | 2.04 | lipase |
| Le3092 | 0 | 0.75 | 0.61 | 0.81 | 0.84 | 0.6 | 1.02 | 1.13 | hydrolase, HAD superfamily |
| Le3112 | 0 | 0.88 | 0.95 | 0.67 | 0.94 | 0.84 | 1.23 | 1.41 | PTS system fructose subfamily transporter subunit IIA |
| Le3169 | 0 | 1.07 | 1.27 | -1.34 | -6.74 | 0.33 | -0.68 | -6.21 | putative comea-related dna uptake protein |
| Le3200 | 0 | 0.71 | 0.64 | 0.68 | 0.43 | 0.63 | 0.89 | 1.07 | hypothetical protein |
| Le3223 | 0 | 0.43 | 0.03 | -0.93 | 0.69 | 0.28 | 0.41 | 1.07 | TonB-dependent receptor |
| Le3239 | 0 | 0.43 | 1.72 | -0.53 | -1.71 | 0.93 | -0.77 | -1.47 | hypothetical protein |
| Le3241 | 0 | 1.37 | 0.93 | 1.32 | 2.02 | -0.69 | 0.59 | 3.08 | conserved hypothetical protein |
| Le3259 | 0 | 0.6 | 0.51 | 0.41 | 0.43 | 1.11 | 1.07 | 1.18 | peptidase |
| Le3307 | 0 | 0.96 | 1.4 | 1.11 | 1.3 | 0.39 | 1.09 | 1.19 | inner membrane protein |
| Le3323 | 0 | 0.36 | 0.4 | 0.14 | 0.39 | 0.5 | 0.29 | 1.05 | transcriptional regulator |
| Le3349 | 0 | 0.54 | 1.54 | 1.8 | 0.67 | 1.6 | 1.37 | 2.11 | PadR family transcriptional regulator |
| Le3353 | 0 | 0.69 | 0.51 | 0.48 | 0.17 | 0.78 | 0.79 | 1.01 | putative membrane protein |
| Le3365 | 0 | 0.91 | 1.09 | 0.6 | 0.33 | 0.61 | 0.94 | 1.07 | hypothetical protein |
| Le3369 | 0 | 1.6 | 1.47 | 0.51 | 1.43 | 1.5 | 2.08 | 2.17 | hypothetical protein |
| Le3373 | 0 | 0.7 | 0.26 | 0.41 | 0.83 | 0.57 | 0.89 | 1.4 | hypothetical protein |
| Le3393 | 0 | 1.22 | 0.74 | 0.97 | 1.12 | 0.87 | 1.14 | 1.44 | hypothetical protein |
| Le3403 | 0 | 0.61 | 0.12 | 0.29 | -0.05 | 1.06 | 0.8 | 1.08 | hypothetical protein |
| Le3421 | 0 | 0.9 | 0.55 | 0.21 | 0.6 | 0.85 | 1.24 | 1.3 | RNA-binding protein Hfq |
| Le3430 | 0 | 1.01 | 1.79 | 0.75 | 1.68 | 1.25 | 1.86 | 1.34 | conserved repeat domain protein |
| Le3437 | 0 | 0.02 | -0.61 | -0.27 | -2.96 | 0.22 | -1.66 | -1.53 | hypothetical protein |
| Le3501 | 0 | 3.02 | 3.06 | 1.85 | 3.45 | 2.4 | 1.98 | 3.62 | membrane protein |
| Le3531 | 0 | 0.74 | 0.87 | 0.09 | 0.34 | 0.66 | 1.04 | 1.12 | hypothetical protein |
| Le3535 | 0 | 0.13 | -0.32 | 0.03 | 0.4 | -0.04 | -0.06 | 1.34 | hypothetical protein |
| Le3547 | 0 | 0.53 | 2.48 | 1.86 | 0.65 | 0.69 | 1.31 | 1.63 | alpha/beta fold family hydrolase |
| Le3561 | 0 | -0.01 | 0.42 | 0.41 | 0.22 | 0.24 | 0.67 | 1.16 | XRE family transcriptional regulator |
| Le3562 | 0 | 1.03 | 0.2 | 0.02 | 0.47 | 1.01 | 1.04 | 1.06 | protein of unknown function DUF1275 |
| Le3588 | 0 | 0.88 | 0.68 | 0.71 | 1.14 | 0.47 | 0.93 | 1.97 | 3-deoxy-D-manno-octulosonic-acid kinase |
| Le3600 | 0 | 0.93 | 0.14 | 0.36 | 0.19 | 1.05 | 0.79 | 1.16 | phage portal protein, PBSX family |
| Le3626 | 0 | 0.63 | 0.96 | 0.93 | 0.86 | 0.8 | 1.17 | 1.88 | hypothetical protein |
| Le3643 | 0 | 0.43 | 0.31 | 0.56 | 0.38 | 0.76 | 0.8 | 1.02 | putative negative regulator of sigma E activity |
| Le3690 | 0 | 0.12 | 0.09 | -0.56 | -1.22 | -0.42 | -1.07 | -1.35 | hypothetical protein |
| Le3691 | 0 | -0.19 | -0.23 | -0.28 | -1.42 | -0.53 | -0.99 | -1.14 | hypothetical protein |
| Le3698 | 0 | 0.92 | 0.49 | -0.09 | -0.18 | 0.91 | 1.07 | 1.02 | hypothetical protein |
| Le3787 | 0 | 0.07 | 1.69 | -0.7 | -1.47 | 0.59 | -0.63 | -1.1 | acyl-CoA synthetase |
| Le3817 | 0 | 3.01 | 2.17 | 2.56 | 2.95 | 2.67 | 3.1 | 3.24 | chitooligosaccharide deacetylase, nodulation protein |
| Le3834 | 0 | 1.1 | 2.01 | 1.54 | 1.99 | 1.68 | 1.65 | 1.5 | hypothetical protein |
| Le3835 | 0 | 1.31 | 1.94 | 1.45 | 2.25 | 2.1 | 2.19 | 1.94 | ATP phosphoribosyltransferase |
| Le3836 | 0 | 1.18 | 1.27 | 1.12 | 2.08 | 2.1 | 2.31 | 2.17 | bifunctional histidinal dehydrogenase/ histidinol dehydrogenase |
| Le3837 | 0 | 0.28 | -0.1 | -0.12 | 0.72 | 1.01 | 1.53 | 1.52 | histidinol-phosphate aminotransferase |
| Le3846 | 0 | 1.3 | 1.55 | 1.16 | 1.24 | 2.26 | 2.31 | 2 | 3-dehydroquinate dehydratase |
| Le3857 | 0 | 0.82 | 0.14 | -0.37 | 0.69 | 0.28 | 0.72 | 1.22 | type 12 methyltransferase |
| Le3873 | 0 | 0.43 | 0.71 | 0.66 | 0.58 | 0.43 | 0.81 | 1.01 | hypothetical protein |
| Le3907 | 0 | 0.78 | 0.97 | 0.09 | 0.6 | 0.85 | 1.06 | 1.25 | hypothetical protein |
| Le3916 | 0 | 4.46 | 4.92 | 4.26 | 5.03 | 3.41 | 3.68 | 6.54 | hypothetical protein |
| Le3962 | 0 | -0.25 | 0.57 | 0.14 | -0.98 | 1.32 | -1.76 | -2.11 | hypothetical protein |
| Le4001 | 0 | 1.14 | 1.41 | 1.07 | 1.01 | 0.69 | 1.28 | 1.77 | phosphotyrosine protein phosphatase |
| Le4029 | 0 | 1.13 | 0.86 | 0.87 | 1.08 | 1.24 | 1.15 | 1.51 | hypothetical protein |
| Le4031 | 0 | 2.03 | -0.21 | 1.64 | 1.69 | -0.13 | 1.56 | 2.41 | hypothetical protein |
| Le4103 | 0 | 0.58 | 0.42 | 0.44 | 0.56 | 0.49 | 1.04 | 1.04 | hypothetical protein |
| Le4112 | 0 | 0.58 | 0.44 | 0.58 | 0.7 | 0.61 | 0.97 | 1.02 | lipopolysaccharide biosynthesis protein |
| Le4153 | 0 | 0.51 | -0.35 | 0.45 | 0.69 | 0.11 | 0.78 | 1.16 | hypothetical protein |
| Le4161 | 0 | 0.9 | 0.37 | 0.39 | 0.43 | 0.7 | 0.71 | 1.04 | hypothetical protein |
| Le4169 | 0 | 0.77 | 0.41 | 0.64 | 1.03 | 1.06 | 1.37 | 1.53 | citrate transporter |
| Le4186 | 0 | 0.88 | 0.68 | 0.33 | 1.1 | 0.37 | 1.15 | 1.26 | protein of unknown function DUF833 |
| Le4209 | 0 | 0.53 | 0.8 | 0.27 | 1 | 0.23 | 0.55 | 1.89 | heme exporter protein CcmD |
| Le4210 | 0 | 1.17 | 0.59 | 0.19 | 0.53 | 1 | 1.43 | 1.18 | heme ABC transporter permease |
| Le4213 | 0 | 0.98 | 0.83 | 0.29 | 0.82 | 0.85 | 1.28 | 1.41 | hypothetical protein |
| Le4269 | 0 | 0.3 | 0.71 | 0.84 | 0.59 | 0.79 | 0.94 | 1.32 | TetR family transcriptional regulator |
| Le4273 | 0 | 0.45 | 0.94 | 0.69 | 0.87 | 0.46 | 0.98 | 1.18 | nucleoside-diphosphate-sugar epimerase |
| Le4275 | 0 | 0.42 | 0.04 | -0.21 | 0.58 | 0.41 | 0.69 | 1 | MFS transporter |
| Le4388 | 0 | 1.69 | 1.99 | 0.72 | -0.71 | 0.05 | -0.1 | 2.6 | hypothetical protein |
| Le4407 | 0 | 0.9 | 0.07 | 0.99 | 0.06 | 1.51 | 1.14 | 1.04 | methyltransferase domain-containing protein |
| Le4411 | 0 | 0.84 | 0.9 | 0.41 | 0.5 | 0.72 | 1.51 | 1.02 | hypothetical protein |
| Le4487 | 0 | 0.98 | 0.4 | 0.53 | 0.36 | 1.15 | 1.21 | 1.19 | hypothetical protein |
| Le4521 | 0 | 1.29 | 1.14 | 0.67 | 0.59 | 0.42 | 1.06 | 1.67 | hypothetical protein |
| Le4530 | 0 | 0.47 | 1.07 | 1.27 | 2.07 | 0.65 | -0.38 | 1.4 | oxidoreductase, FAD-binding protein |
| Le4549 | 0 | 3.19 | 2.32 | 2.26 | 2.1 | 4.22 | 4.24 | 4.73 | hypothetical protein |
| Le4554 | 0 | 1.61 | 2.68 | 1.08 | 1.39 | 1.87 | 1.86 | 1.72 | capsular polysaccharide biosynthesis protein |
| Le4582 | 0 | 0.74 | -0.08 | 0.3 | 0.12 | 1 | 1.37 | 1.46 | unknown |
| Le4592 | 0 | 0.56 | 0.53 | 1.18 | 0 | -0.11 | 0.38 | 1.97 | hypothetical protein |
| Le4599 | 0 | -0.07 | 0.16 | -0.3 | -0.8 | -0.11 | 0.39 | -2.27 | alpha/beta hydrolase fold protein |
| Le4600 | 0 | 1.03 | 1.59 | 1.31 | 1.52 | 1.09 | 1.22 | 1.78 | putative transcriptional regulator |
| Le4610 | 0 | 1.22 | 0.35 | 0.77 | 1.43 | 1.83 | 1.67 | 1.65 | sec-independent protein translocase protein TatC |
| Le4612 | 0 | 1.42 | 0.91 | 0.97 | 0.83 | 1.07 | 1.07 | 1.63 | ranscriptional regulator, BadM/Rrf2 family |
| Le4624 | 0 | 0.36 | 0.4 | 0.11 | -0.05 | 0.36 | 0.35 | 1.03 | alkaline phosphatase |
| Le4627 | 0 | -0.26 | 0.36 | -0.14 | -1.38 | -0.16 | -1.13 | -1.39 | Pyridoxal-dependent decarboxylase |
| Le4660 | 0 | 0.66 | -0.18 | 0.45 | 1 | 0.71 | 1.32 | 1.34 | acyl-CoA dehydrogenase |
| Le4683 | 0 | 0.98 | 0.82 | 0.18 | 0.14 | 1.05 | 1.16 | 1.06 | hypothetical protein |
| Le4693 | 0 | 0.99 | 0.37 | 0.86 | 1.32 | 1.28 | 1.09 | 1.4 | probable leucyl/phenylalanyl-tRNA--protein transferase (leucyltransferase) |
| Le4695 | 0 | -0.38 | 0.77 | -0.28 | -1.94 | 0.64 | -0.7 | -2.2 | hypothetical protein |
| Le4738 | 0 | 0.56 | 0.17 | 0.86 | 1.11 | 0.83 | 1.15 | 1.13 | alpha-1 2-mannosidase |
| Le4739 | 0 | 0.33 | 0 | 0.58 | 0.93 | 0.62 | 1.01 | 1.07 | outer membrane receptor for ferrienterochelin and colicins |
| Le4749 | 0 | 0.06 | -0.55 | 0.14 | 1.02 | -0.09 | 0.73 | 1.39 | hypothetical protein |
| Le4792 | 0 | 0.17 | 0.27 | -0.11 | 0.27 | 0.22 | 0.79 | 1.16 | hypothetical protein |
| Le4795 | 0 | 0.33 | 0.89 | 0.2 | 0.51 | 0.46 | 0.38 | 1.18 | hypothetical protein |
| Le4798 | 0 | 0.53 | 0.29 | -0.19 | 0 | 0.55 | 0.94 | 1.59 | putative exported protein |
| Le4808 | 0 | 0.28 | 0.71 | 0.34 | 0.58 | -0.48 | 0.85 | 1.44 | hypothetical protein |
| Le4819 | 0 | -0.01 | -0.4 | 0.48 | 1.35 | -0.21 | 0.36 | 1.32 | DUF1800 domain-containing protein |
| Le4820 | 0 | 0.04 | -0.17 | 0.47 | 1.36 | 0.01 | 0.85 | 1.41 | hypothetical protein |
| Le4892 | 0 | 1.7 | 2.12 | 0.49 | 0.6 | 1.61 | 1.31 | 1.12 | hypothetical protein |
| Le4895 | 0 | 0.68 | 0.33 | 0.44 | 0.49 | 0.47 | 0.84 | 1.15 | thioesterase |
| Le4896 | 0 | 0.62 | 0.6 | 0.35 | 1.15 | 0.85 | 1.65 | 1.57 | Acriflavin resistance protein |
| Le4897 | 0 | 0.74 | 0.74 | 0.69 | 1.44 | 1.14 | 1.82 | 1.88 | efflux RND transporter periplasmic adaptor subunit |
| Le4898 | 0 | 0.41 | 0.35 | 0.13 | 1.1 | 0.64 | 1.42 | 1.37 | diaminobutyrate--2-oxoglutarate aminotransferase |
| Le4899 | 0 | 0.26 | 0.39 | 0.11 | 1.03 | 0.61 | 1.46 | 1.3 | hypothetical protein |
| Le4900 | 0 | 0.3 | 0.44 | 0.43 | 1.03 | 0.65 | 1.46 | 1.2 | hypothetical protein |
| Le4901 | 0 | 0.1 | 0.15 | 0.33 | 1 | 0.48 | 1.37 | 1.13 | ABC-type multidrug transport system, ATPase component |
| Le4902 | 0 | 0.43 | 0.5 | 0.67 | 1.58 | 0.78 | 1.97 | 1.79 | hypothetical protein |
| Le4903 | 0 | 0.24 | 0.22 | 0.77 | 1.63 | 0.64 | 2.02 | 1.7 | hypothetical protein |
| Le4904 | 0 | 0.09 | 0.21 | 0.65 | 1.63 | 0.56 | 1.91 | 1.62 | glycine C-acetyltransferase |
| Le4905 | 0 | 0.24 | 0.25 | 0.69 | 1.83 | 0.67 | 2.01 | 1.78 | hypothetical protein |
| Le4906 | 0 | 0.45 | 0.44 | 0.9 | 2.02 | 0.92 | 2.27 | 2.08 | NAD-dependent epimerase |
| Le4907 | 0 | 0.58 | 0.55 | 0.71 | 1.6 | 0.85 | 1.95 | 1.77 | hypothetical protein |
| Le4920 | 0 | 0.79 | -0.19 | -0.33 | 1.82 | 0.18 | 0.78 | 1.45 | putative autotransporter protein, putative Ig domain-containing protein |
| Le4930 | 0 | 0.53 | 0.51 | 0.7 | 0.82 | 0.58 | 0.78 | 1.43 | alcohol dehydrogenase |
| Le4931 | 0 | 0.73 | 0.71 | 0.82 | 0.83 | 1.26 | 1.42 | 1.33 | MFS transporter |
| Le4951 | 0 | 0.91 | 0.62 | 1.03 | 1.25 | 1.14 | 1.46 | 1.51 | hypothetical protein |
| Le4956 | 0 | 0.88 | 1.76 | 1.88 | 0.9 | 0.7 | 1.21 | 1.93 | peptidase S41 |
| Le4976 | 0 | 1 | 0.79 | 0.59 | 0.94 | 1.27 | 1.78 | 1.6 | hypothetical protein |
| Le5041 | 0 | -10.93 | -4.7 | -6.02 | -10.93 | -4.43 | -4.6 | -5.6 | transcriptional regulator |
| Le5059 | 0 | 0.45 | 0.8 | 0.3 | 1.09 | -0.23 | -0.13 | 1.76 | ferredoxin |
| Le5089 | 0 | 0.55 | 0.83 | 0.36 | 0.91 | 1.22 | 1.11 | 1.02 | conserved hypothetical protein |
| Le5091 | 0 | 1.44 | 1.73 | 1.2 | 1.72 | 2.45 | 2.4 | 1.77 | potassium-transporting ATPase subunit B |
| Le5109 | 0 | 0 | -0.36 | -0.75 | -3.73 | -0.25 | -3.21 | -2.34 | arginase-like protein |
| Le5111 | 0 | -0.36 | -0.66 | -1 | -3.92 | -0.42 | -3.75 | -2.76 | ferredoxin reductase-like protein |
| Le5112 | 0 | -0.19 | -0.55 | -1.07 | -4.37 | -0.46 | -4.18 | -3.26 | sterol desaturase-like protein |
| Le5113 | 0 | -0.18 | -0.38 | -0.89 | -3.92 | -0.15 | -3.42 | -2.82 | hybrid polyketide synthase and nonribosomal peptide synthetase |
| Le5114 | 0 | -0.14 | -0.36 | -0.95 | -3.87 | -0.06 | -3.27 | -2.79 | Ox1, FAD dependent oxidoreductase |
| Le5115 | 0 | -0.09 | -0.36 | -0.92 | -3.76 | -0.04 | -3.22 | -2.73 | Ox2, FAD dependent oxidoreductase |
| Le5116 | 0 | -0.15 | -0.43 | -0.96 | -3.79 | -0.13 | -3.22 | -2.81 | Ox3, FAD dependent oxidoreductase |
| Le5117 | 0 | -0.09 | -0.4 | -0.94 | -3.84 | -0.14 | -3.24 | -2.73 | Ox4, FAD dependent oxidoreductase |
| Le5118 | 0 | -0.06 | -0.15 | -0.79 | -3.47 | 0.08 | -3.13 | -2.43 | membrane transporter |
| Le5119 | 0 | 0.09 | -0.22 | -0.86 | -3.57 | 0.03 | -2.84 | -2.55 | hypothetical protein |
| Le5120 | 0 | -0.08 | -0.35 | -1.07 | -3.55 | -0.13 | -2.96 | -2.79 | trypsin-like peptidase domain-containing protein |
| Le5121 | 0 | 0.34 | 0.12 | -1.16 | -2.38 | 0.25 | -2.09 | -2.67 | M15 family metallopeptidase |
| Le5151 | 0 | 0.42 | 0.14 | 0.33 | -0.14 | 0.95 | 0.69 | 1.03 | membrane protein |
| Le5250 | 0 | 0.84 | 0.76 | 0.83 | 0.9 | 0.68 | 1.1 | 1.29 | zinc transporter |
